# Challenges in differential diagnosis between autism spectrum disorder and psychotic disorders

**DOI:** 10.1192/j.eurpsy.2023.100

**Published:** 2023-07-19

**Authors:** K. Krysta

**Affiliations:** Department of Rehabilitation Psychiatry, Medical University of Silesia, Katowice, Poland

## Abstract

**Abstract:**

Data from literature show that people with Autism Spectrum Disorders (ASD) are more likely to develop a psychosis than the general population. They also have a higher chance of developing schizophrenia than neurotypical controls. In order to diagnose comorbid psychosis in ASD in an adult, first a full psychiatric examination is necessary to decide on the directions of possible further diagnostics and, if necessary, to recommend appropriate additional tests, e.g. psychological tests using professional scales and questionnaires for adults. In the case of diagnostic doubts, there are indications to refer the patient to more specialized centers. Although the two conditions are different, they share some common features, such as social withdrawal and communication disorders. This can lead to problems in the diagnosis of psychosis in people with autism. The basic symptoms of the mentioned diseases usually differ between the two disorders. For this reason, it’s important to try to get regular screenings and get care from the right professionals. A case report is presented of a patient, who received a diagnosis of ASD in the childhood, and later as an adult developed psychotic symptoms, which led to a change of diagnosis to schizophrenia.

**Disclosure of Interest:**

None Declared

